# Improving Ammonium and Nitrate Release from Urea Using Clinoptilolite Zeolite and Compost Produced from Agricultural Wastes

**DOI:** 10.1155/2015/574201

**Published:** 2015-02-22

**Authors:** Latifah Omar, Osumanu Haruna Ahmed, Nik Muhamad Ab. Majid

**Affiliations:** ^1^Department of Crop Science, Faculty of Agriculture and Food Sciences, Universiti Putra Malaysia, Bintulu Sarawak Campus, 97008 Bintulu, Sarawak, Malaysia; ^2^Agriculture and Environment, Borneo Ecosystem Science Research Centre, Faculty of Agriculture and Food Sciences, Universiti Putra Malaysia, Bintulu Sarawak Campus, 97008 Bintulu, Sarawak, Malaysia; ^3^Institute of Tropical Forestry and Forest Product (INTROP), Universiti Putra Malaysia, 43400 Serdang, Selangor, Malaysia

## Abstract

Improper use of urea may cause environmental pollution through NH_3_ volatilization and NO_3_^−^ leaching from urea. Clinoptilolite zeolite and compost could be used to control N loss from urea by controlling NH_4_^+^ and NO_3_^−^ release from urea. Soil incubation and leaching experiments were conducted to determine the effects of clinoptilolite zeolite and compost on controlling NH_4_^+^ and NO_3_^−^ losses from urea. Bekenu Series soil (*Typic Paleudults*) was incubated for 30, 60, and 90 days. A soil leaching experiment was conducted for 30 days. Urea amended with clinoptilolite zeolite and compost significantly reduced NH_4_^+^ and NO_3_^−^ release from urea (soil incubation study) compared with urea alone, thus reducing leaching of these ions. Ammonium and NO_3_^−^ leaching losses during the 30 days of the leaching experiment were highest in urea alone compared with urea with clinoptilolite zeolite and compost treatments. At 30 days of the leaching experiment, NH_4_^+^ retention in soil with urea amended with clinoptilolite zeolite and compost was better than that with urea alone. These observations were because of the high pH, CEC, and other chemical properties of clinoptilolite zeolite and compost. Urea can be amended with clinoptilolite zeolite and compost to improve NH_4_^+^ and NO_3_^−^ release from urea.

## 1. Introduction

Depending on soil pH, moisture, and application methods, urea undergoes chemical transformation to produce either NH_4_^+^ or NO_3_^−^ [1]. Nitrogen from urea is subject to loss from a number of pathways of which leaching of NO_3_^−^ is one of the most important pathways because NO_3_^−^ is extremely mobile. Leaching of NO_3_^−^ from urea leads to increase in NO_3_^−^ concentrations in surface and ground water [2]. Leaching losses of N occur when soils have more incoming water than they can hold. As water moves through the soil, NO_3_^−^ in the soil solution moves along with the water. Because NH_4_^+^ is positively charged, it is held by the negative sites of soils (e.g., clay and humus); therefore, NH_4_^+^ leaches less in mineral soils which are particularly high in clay [3]. In contrast, NH_4_^+^ leaching is significant in coarse-textured sands and some muck soils [4]. Thus, agricultural systems research leading to management practices that improve N utilization efficiency and decrease N losses is essential [5].

Nitrogen leaching loss in soils is a risk because if NO_3_^−^ is not absorbed by the plant root system, it is leached below the root zone of plants, thus contaminating groundwater [6]. According to Paramasivam et al. [7], optimization of irrigation and avoidance of fertilization during rainy seasons could minimize leaching loss of N. However, this approach can be difficult to achieve because rapidly growing crops require adequate N fertilizer. Leaching loss of N can be reduced by minimizing the amounts of NH_4_^+^ and NO_3_^−^ in soils. However, this is a challenge because it requires simultaneous management of N fertilizers and water. Loss of mineral N from agricultural systems is difficult to achieve through reduction of N fertilizers use [8]. One of the better methods of reducing losses of mineral N is the use of clinoptilolite zeolite.

Clinoptilolite zeolite can be used to control N loss from urea because of the small molecular size of the open-ringed structure of clinoptilolite zeolite which physically protects NH_4_^+^ ions against microbial nitrification [9]. Clinoptilolite zeolite is a mineral with a unique structure which allows entrapping or releasing various cations due to its high cation exchange capacity [10]. Adoption of management techniques such as clinoptilolite zeolite utilization, which maximizes N use efficiency and water use efficiency, may decrease the excessive and unbalanced use of N fertilizers in agriculture. Unbalanced use of N fertilizers could cause environmental pollution [11]. Studies have shown that the use of clinoptilolite zeolite and N fertilizers improves N use efficiency [12–15]. The increased efficiency of N utilization when urea is used together with clinoptilolite zeolite has been demonstrated by a number of researchers [16–18].

As the third largest producer of chicken products in Asia, chicken manure disposal is becoming a challenge in Malaysia. In 2012, 674 million and 637 million day-old chicks and broilers, respectively, were produced [19]. Wastes generated in the poultry farms are increasing as the poultry farming grows. The daily manure production by a laying hen has been estimated as 138 g day^−1^ (25% dry substance) and 90 g day^−1^ (40% dry substance) by a broiler [20]. Large quantities of rice straw are produced every harvesting season in Malaysia. Managing this waste in Malaysia is a challenge. In Malaysia, there is 684,000 ha of paddy fields from which 1.3 million tonnes of rice straw is produced every year [21]. Cocomposting these wastes to produce organic amendments such as compost is essential.

Cocomposting is a simple method which converts, for example, chicken manure and rice straw into valuable organic amendments. The process of cocomposting is essential for aforementioned wastes to be safely, conveniently, and efficiently used as soil organic amendment [22]. This is because during cocomposting, a large part of the original organic matter is mineralized and the residual organic matter is transformed into new organic materials (e.g., humic-like substances such as humic and fulvic acids). Application of compost in agriculture is very desirable worldwide as compost is most often used to improve soil structure and content of soil organic matter [23]. However, the effects of compost addition to soil on the fate of mineral N are scarcely studied. For example, there is dearth of information on improving NH_4_^+^ and NO_3_^−^ release from urea using clinoptilolite zeolite and composts. Thus, the objective of this study was to determine the effects of clinoptilolite zeolite and compost (produced by cocomposting rice straw and chicken manure) on controlling NH_4_^+^ and NO_3_^−^ loss from urea.

## 2. Materials and Methods

### 2.1. Selected Chemical Properties of Soil, Clinoptilolite Zeolite, and Compost

The soil used in this study was Bekenu Series (*Typic Paleudults*). The soil was sampled at 0–20 cm depth from an uncultivated area at Universiti Putra Malaysia, Bintulu Campus Sarawak, Malaysia. The soil was air-dried and ground to pass a 2.0 mm sieve for initial characterization, incubation, and leaching experiments. Field capacity and bulk density of the soil were determined by the method described by Tan [26]. Soil field capacity was measured using a graduated cylinder. This procedure involves calculation of the water percolate in a measuring cylinder. A 10 g soil was weighted into a funnel, the base of it filled with Whatman filter paper number 2 to avoid losing of soil. Afterwards, a 100 mL of distilled water was poured onto the soil in cylinder and let to drain. All the water drained from the soil by gravity was considered the field capacity of the soil [26]. Soil bulk density was determined using the core ring method [26]. The core ring was placed with the sharpened side down, on top of the soil after which the core ring was gently hammered into the soil with hammer. Afterwards, a piece of hard wood was placed over the core ring until the top of it was flushed with the soil surface. A scoop was used to dig a small trench on one side of the core ring. It was slightly deeper than the ring. Afterwards, the soil sample with the core ring was placed in an oven at temperature of 105°C for 24 hours and cooled in a desiccator. The bulk density was calculated considering the mass of oven dry soil, soil volume, diameter, weight, and height of ring sampler, as well as weight of soil and ring sampler [26].

Soil texture was determined using the hydrometer method [27]. The hydrometer method determines the texture of soil by measuring total sand (2.0–0.05 mm), silt (0.05–0.002 mm), and clay (<0.002 mm). A 50 g of soil sample was weighed and placed in a blender cup. Distilled water was added to the sample within 10 cm of the top and mixed with 4 drops of 3 M NaOH. The mixture was stirred continuously until the soil suspension had a pH of 10. Then the mixture was stirred mechanically for 15 minutes, after which it was transferred into a 1000 mL measuring cylinder. The remaining soil residue in a blender cup was sprayed with distilled water until volume of the sample in a 1000 mL measuring cylinder was made up to 1130 mL. The soil suspension was stirred continuously for a homogenous mixture. Afterwards, a hydrometer was used to measure first reading after 40 seconds. The suspension was stirred six times and after 40 seconds, the hydrometer was inserted in the suspension to obtain second reading. The suspension temperature was recorded after removing the hydrometer from the suspension. Average of two readings gave the amount of silt and clay in grams of the soil sample. The suspension was stirred thoroughly after 2 hours of settling time and the third reading was obtained to get the amount of clay in grams. The temperature of the suspension was recorded shortly after the hydrometer was dipped into the suspension for every reading because the hydrometer was calibrated at 20°C [27].

The pH of the soil was determined in a ratio of 1 : 2 (soil : distilled water suspension) using a digital pH meter [28]. A 10 g of soil was weighed and placed in plastic vials. Distilled water of 20 mL was added and shaken at 180 rpm for 15 minutes [28]. The reading for pH in water was recorded after 24 hours. A digital pH meter (Seven Easy Mettler Toledo) was used to record pH reading. Soil organic matter, C, and N were determined using LECO CHNS Analyzer (LECO Truspec Micro Elemental Analyzer CHNS, New York). A 2.2 mg ground soil (250 *μ*m) was placed on the loading head of the machine. Samples were combusted at 1075°C and the reading of total C and N was obtained directly from computer software. The loss of weight represented the weight of C and organic matter content was estimated by multiplying organic matter value; 58% of organic matter is C [29]. Soil available P was extracted using the double acid method [26] followed by the blue method [30]. A 5 g of soil was weighed and placed in a 250 mL Erlenmeyer flask. A 20 mL extracting solution was added and shaken mechanically at 180 rpm for 10 minutes. Afterwards, the supernatant was filtered using Whatman filter paper number 2.

Acid molybdate stock solution (Reagent A) and ascorbic acid stock solution (Reagent B) were prepared for colour development procedure. A standard P solution (standard solution 1) and standard solution 2 were prepared and used to prepare working solutions ranging from 0 to 0.6 ppm. 0, 1, 2, 3, 4, 5, and 6 mL of standard solution 2 were pipetted into 50 mL volumetric flask using micropipette and added with 8 mL of Reagent B to develop the blue colour. A 4 mL of the supernatant was pipetted into 50 mL volumetric flask. Reagent B of 8 mL was added to develop the blue colour. This solution was diluted to mark with distilled water and maximum blue colour was allowed to develop [30]. The absorbance was measured at 840 nm. Standard curve was prepared by pipetting 0, 1, 2, 3, 4, 5, and 6 mL of the P standard solution into 7 series of 50 mL volumetric flasks. Samples including standard solution were read using UV-VIS spectrophotometer (Perkin Elmer Lambda 25, USA).

Exchangeable cations were extracted using the leaching method [26]. A 10 g soil was placed in a leaching tube and leached with 100 mL NH_4_OA_c_ solution for 5 hours. Their concentrations in the extract were determined using Atomic Absorption Spectrophotometry (Analyst 800, Perkin Elmer, Norwalk, USA). Soil CEC was determined by leaching the soil samples with 100 mL of 1 M NH_4_OA_c_ for 5 hours [26]. Afterwards, the soil sample was washed with 30 mL of 95% ethanol. The leachate was collected in a 100 mL volumetric flask and distilled water was added to make up volume of a 100 mL volumetric flask. A 10 mL of sample from leachate was pipetted into distillation flask and added with 10 mL of 40% NaOH. The samples were distilled for 5 minutes and collected with 10 mL of 2% boric acid with bromocresol green and methyl red indicator [31]. Then, the distillate was titrated with 0.01 M HCl [26]. The method of Keeney and Nelson [32] was used to extract exchangeable NH_4_^+^ and available NO_3_^−^ after which their concentrations were determined using steam distillation. A sample of 5 g soil was extracted with 50 mL of 2 M KCl solution and shaken for 1 hour. Afterwards, it was filtered using Whatman filter paper number 2. Exchangeable NH_4_^+^ was determined by pipetting 20 mL of extractant into distillation tube, added with 0.2 g of MgO, and distilled for 5 min. A 10 mL of boric acid with bromocresol green and methyl red indicator was used to trap NH_4_^+^. Afterwards, the distillation was continued by adding 0.2 g Devarda's alloy to the sample and the process of distillation proceeded for another 5 minutes. The distillates of NH_4_^+^ and NO_3_^−^ were titrated with 0.01 M HCl. The texture of the soil was sandy loam with a bulk density of 1.51 g m^−3^. The texture of the soil and bulk density are consistent with those reported in Soil Survey Staff [33]. The selected chemical properties of the soil are summarized in Table 1. The soil pH, total N, and total C were consistent with those reported for Bekenu Series [24]. The exchangeable Ca, Mg, and K in this study were higher than the reported values [24].

The clinoptilolite zeolite used in this study was in powder form. Total N of the clinoptilolite zeolite was determined using Kjeldahl method [31]. 0.5 g of clinoptilolite zeolite was mixed with 5 mL concentrated sulfuric acid (H_2_SO_4_) and digested. A tablet of selenium catalyst was added, shaken, and left to equilibrate for 30 minutes. The samples were then digested at 180°C for 1 h and then at 320°C for 4 to 5 h until samples were colourless. 10 mL of the digested sample was pipetted into 50 mL distillation flask and added with 10 mL 40% NaOH. 10 mL of boric acid plus indicator solution was placed at the end of the condenser in the distillation apparatus to trap NH_3_ released. The mixture was distilled until the boric acid-indicator solution doubled the original volume. The distillate solution was titrated with 0.01 M HCl to estimate N content in the clinoptilolite zeolite. The pH, exchangeable NH_4_^+^, and available NO_3_^−^ of the clinoptilolite zeolite were determined using the method described previously [28, 32]. The CEC of the clinoptilolite zeolite was determined using the CsCl method [34]. The procedure is similar to the procedure of soil CEC determination as outlined previously but 1 M of CsCl instead of 1 M NH_4_OA_c_ was used. The CsCl method was used to avoid underestimation of CEC of the clinoptilolite zeolite as the method does not lead to entrapment of ammonium ions in the channels of the clinoptilolite zeolite. The exchangeable K, Ca, and Mg of the clinoptilolite zeolite were extracted using the method of Ming and Dixon [34] and their concentrations determined using Atomic Absorption Spectrophotometry (Analyst 800, Perkin Elmer, Norwalk, USA). The chemical composition of the clinoptilolite zeolite used in this study is summarized in Table 2.

The selected chemical characteristics of the compost were determined using standard procedures as outlined previously [26–32]. The selected chemical properties of the compost produced by cocomposting rice straw and chicken manure are summarized in Table 3. Values were obtained from our previous study on cocomposting rice straw and chicken manure (paper under review). Total N of the compost was 1.80% (Table 3). Carbon to N ratio of the compost was 15.17. Humic acid content, ash content, NH_4_^+^, NO_3_^−^ P, Ca, Mg, and K concentrations increased after the cocomposting process (Table 3). The lower concentrations of Cu, Fe, Mn, Zn, and microbial biomass of the compost suggest that the compost is stable, mature, and not toxic. The seeds germination rate of maize (*Zea mays* L.) tested on compost and distilled water (control) using the method described by Zucconi et al. [35] and spinach (*Spinacia oleracea*) growth on peat-based growing medium (control) [36] and compost was not significantly different, suggesting that the compost is not phytotoxic.

The humic acid fractions after the composting process were extracted with 0.5 M NaOH, precipitated at pH 1.0 with 6 M HCl, and then purified with 100 mL distilled water [37]. The carboxyl and phenolic contents of the humic acid extracted from the compost were determined according to the method described by Stevenson [38]. 20 mg of humic acid was dissolved in 4 mL of 0.08 M NaOH and shaken for 30 minutes at 180 rpm. The solution was titrated against 0.01 M HCl to pH 2.5. Phenolic content was determined based on the amount of acid required to titrate the solution from pH 10 to pH 8 and it was estimated that 50% of the phenolic group dissociates from pH 10 to pH 8 [39]. Carboxylic content was calculated based on the amount of acid needed to titrate the solution from pH 8 to pH 2.5 and the total acidity was calculated by the summation of carboxylic and phenolic content [39]. To obtain the E_4_/E_6_ ratio of the humic acid, the absorbance at 465 nm and 665 nm was measured using UV-Vis spectrophotometer (Perkin Elmer Lambda 25, USA) on solution of 3 mg of each humic acid in 10 mL of 0.05 M NaHCO_3_. The ratio of absorbance at 465 nm and 665 nm gave the E_4_/E_6_ ratio [25]. The selected chemical properties of the humic acid extracted from the compost are shown in Table 4.

### 2.2. Soil Incubation Experiment

A soil incubation experiment was carried out for 90 days in the Soil Science Laboratory of Universiti Putra Malaysia, Bintulu Sarawak Campus, Malaysia. The treatments evaluated in this experiment were

- 250 g soil (no urea) (T0),
- 250 g soil + 7.40 g urea without additives (T1),
- 250 g soil + 7.40 g urea + 6 g clinoptilolite zeolite (T2),
- 250 g soil + 7.40 g urea + 6 g compost (T3),
- 250 g soil + 7.40 g urea + 6 g clinoptilolite zeolite + 6 g compost (T4).

The rates of urea [40], clinoptilolite zeolite [41], and compost were scaled down from the standard fertilizer recommendation for* Zea mays* L. cultivation [42]. The soil, urea, clinoptilolite zeolite, and compost used in the incubation experiment were thoroughly mixed manually. Beakers (500 mL) with treatments were sealed with a parafilm. The parafilm was perforated to enable good aeration. The samples were incubated for 30 days, 60 days, and 90 days at 26°C, respectively. Each treatment had 3 replications (i.e., 15 samples for 30 days of incubation, 15 samples for 60 days of incubation, and 15 samples for 90 days of incubation). At 30 days, 60 days, and 90 days of incubation (DAI), the soil samples were air-dried and analyzed for pH in water [28], total N using the Kjeldahl method [31], whereas NH_4_^+^ and NO_3_^−^ contents were determined using the method of Keeney and Nelson [32].

### 2.3. Soil Leaching Experiment

The soil leaching experiment comprised the same treatments as in the incubation experiment. All treatments were thoroughly mixed and scaled down to the amount of 64 g soil. The mixture was then filled in leaching tubes and leached with distilled water after which the leachates were collected at three-day interval based on five-year rainfall data obtained from the Sarawak Meteorological Department, Malaysia [43]. Afterwards, the leachates were analyzed for NH_4_^+^ and NO_3_^−^ using the method of Keeney and Nelson [32] whereas the pH of the leachates was determined using a digital pH meter (Seven Easy Mettler Toledo). The volume of the distilled water used was based on rainy days over 30 days. The volume of the distilled water used every three days in the leaching experiment was 36 mL. The soil samples at 30 days of the leaching experiment were analyzed for total N, exchangeable NH_4_^+^, and available NO_3_^−^ using the standard procedures as outlined previously [31, 32].

### 2.4. Statistical Analysis

The soil incubation experiment was a split-block experimental design in triplicate with two factors, namely, time of incubation (30 days, 60 days, and 90 days) and treatments (soil alone, soil + urea, soil + urea + clinoptilolite zeolite, soil + urea + compost, and soil + urea + clinoptilolite zeolite + compost). The experimental design of the soil leaching experiment was completely randomized design (CRD) with three replications. Analysis of variance (ANOVA) was used to detect treatment effects whereas Tukey's test was used to compare treatment means at *P* ≤ 0.05. The Statistical Analysis System version 9.2 was used for the statistical tests.

## 3. Results and Discussion

### 3.1. Soil Incubation Study

Days of incubation significantly affected total N, exchangeable NH_4_^+^, and available NO_3_^−^ but did not affect soil pH at 30 DAI, 60 DAI, and 90 DAI (Table 5).

### 3.2. Soil Total Nitrogen and pH as Affected by Treatments and Period of Incubation

**Figure 1:**
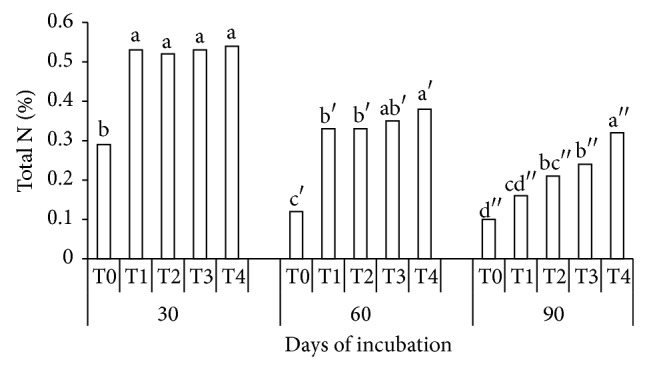
Effects of treatments (T0, T1, T2, T3, and T4) and periods (30, 60, and 90 days) of incubation on soil total N.* Note*. T0: 250 g soil (no urea); T1: 250 g soil + 7.40 g urea (no additives); T2: 250 g soil + 7.40 g urea + 6 g clinoptilolite zeolite; T3: 250 g soil + 7.40 g urea + 6 g compost; T4: 250 g soil + 7.40 g urea + 6 g compost + 6 g clinoptilolite zeolite. Means with the same letter are not significantly different by Tukey's test at *P* ≤ 0.05.* Note*. Letters without prime represent 30 DAI, single prime superscript represents 60 DAI, and double prime superscript represents 90 DAI.

The effects of urea without additives (T1) and urea with additives (T2, T3, and T4) on total N at 30 DAI were not significantly different (Figure 1). However, total N in the soil was significantly higher in T4 compared with T0 and T1 at 60 DAI. Soil total N was significantly higher in T3 and T4 compared with urea without additives (T1) at 90 DAI (Figure 1). These results suggest that mixing urea with soil resulted in greater urea mineralization at 30 DAI and 60 DAI, but urea mixed with compost (T3) and the combination of clinoptilolite zeolite and compost (T4) were more effective in retaining N at 90 DAI. The higher total N in T3 and T4 as compared with T1 at 90 DAI is consistent with the higher organic matter (47.09%) of the compost used in this study (Table 2). This is possible because, in composts, N is present in the form of stable organic N which is slowly but steadily released over time through mineralization. Mineralization of organic N in composts involves the conversion of organic forms of N to NH_3_ or NH_4_^+^ and NO_3_^−^ [1].

The retention of soil total N in T4 could also be due to absorption of NH_4_^+^ and NO_3_^−^ into the clinoptilolite zeolite lattice [9, 16]. This is possible because of the high CEC (100 cmol_c_ kg^−1^) of the clinoptilolite zeolite used in this study. According to Ferguson and Pepper [9], zeolites decreased N concentration in soil solution by trapping NH_4_^+^ through cation exchange. Besides retaining large quantities of NH_4_^+^, these minerals also interfere with the process of nitrification [9, 16].

**Figure 2:**
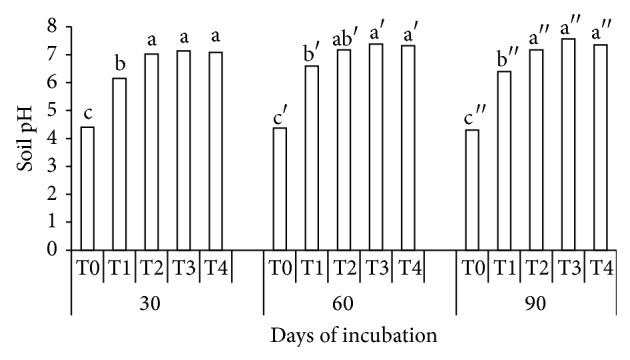
Effects of treatments (T0, T1, T2, T3, and T4) and periods (30, 60, and 90 days) of incubation on soil pH.* Note*. T0: 250 g soil (no urea); T1: 250 g soil + 7.40 g urea (no additives); T2: 250 g soil + 7.40 g urea + 6 g clinoptilolite zeolite; T3: 250 g soil + 7.40 g urea + 6 g compost; T4: 250 g soil + 7.40 g urea + 6 g compost + 6 g clinoptilolite zeolite. Means with the same letter are not significantly different by Tukey's test at *P* ≤ 0.05.* Note*. Letters without prime represent 30 DAI, single prime superscript represents 60 DAI, and double prime superscript represents 90 DAI.

Urea with clinoptilolite zeolite (T2) significantly increased soil pH compared with those of soil alone (T0) and urea without additives (T1) at 30 DAI and 90 DAI but not at 60 DAI (Figure 2). The soil pH was higher in T3 (urea mixed with compost) and T4 (urea mixed with clinoptilolite zeolite and compost) compared with T0 (soil alone) and T1 (urea alone) at 30 DAI, 60 DAI, and 90 DAI, respectively. The higher pH in T2, T3, and T4 was because soil pH increases with urea hydrolysis. As urea hydrolyzes, NH_4_^+^, OH^−^, and CO_3_^2−^ ions are released to increase soil pH [44]. The pH of the compost (7.66) may have partly contributed to the increase in soil pH (Figure 2). Conversion of organic N to NH_4_^+^ for instance is very slow when pH is in the range of 5 to 6 [45]. According to Weier and Gilliam [45], nitrification decreases when soil pH is low (pH 5 to 6). This explains the lower total N in T0 and T1 at 30, 60, and 90 DAI (Figure 1).

### 3.3. Effects of Treatments and Period of Incubation on Soil Exchangeable Ammonium and Available Nitrate in Soil Incubation Experiment

**Figure 3:**
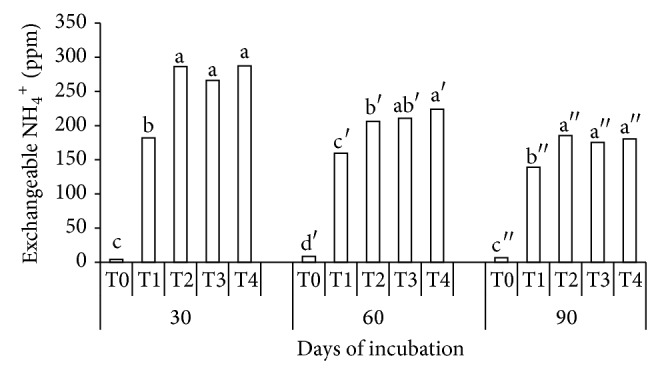
Effects of treatments (T0, T1, T2, T3, and T4) and periods (30, 60, and 90 days) of incubation on soil exchangeable ammonium.* Note*. T0: 250 g soil (no urea); T1: 250 g soil + 7.40 g urea (no additives); T2: 250 g soil + 7.40 g urea + 6 g clinoptilolite zeolite; T3: 250 g soil + 7.40 g urea + 6 g compost; T4: 250 g soil + 7.40 g urea + 6 g compost + 6 g clinoptilolite zeolite. Means with the same letter are not significantly different by Tukey's test at *P* ≤ 0.05.* Note*. Letters without prime represent 30 DAI, single prime superscript represents 60 DAI, and double prime superscript represents 90 DAI.

Soil exchangeable NH_4_^+^ was significantly higher in the urea with clinoptilolite zeolite and compost treatments (T2, T3, and T4) than in urea without additives (T1) and soil alone (T0) at 30 DAI, 60 DAI, and 90 DAI, respectively (Figure 3). The higher concentrations of soil exchangeable NH_4_^+^ in T2, T3, and T4 were partly due to increase in the pH of the soil (Figure 2) as mineralization of organic N to NH_4_^+^ is enhanced by the higher pH [46]. This observation could be one of the reasons why the soil total N of T2, T3, and T4 decreased with increasing period of incubation (Figure 1). It is also possible that some of the NH_4_^+^ released during urea hydrolysis were adsorbed onto the exchange surfaces of clinoptilolite zeolite (T2 and T4) and humic substances of compost (T3 and T4). The retention of soil exchangeable NH_4_^+^ could be attributed to the high CEC of the clinoptilolite zeolite. According to Kithome et al. [47], NH_4_^+^ retained by clinoptilolite zeolite is generally released slowly because of the CEC of this zeolite and nitrification in the soil. Retardation of nitrification in this study may have occurred due to clinoptilolite zeolite, as the small molecular size of the open-ringed structure of clinoptilolite zeolite (10^−6^–10^−9^ m) physically protects NH_4_^+^ against microbial nitrification [9]. The absorption of soil exchangeable NH_4_^+^ in urea with compost treatments (T3 and T4) was possible because of the humic acid content (15.20%) of the compost used in this study. The carboxyl, phenol, and total acidity of the humic acid of the compost used in this study were 450 cmol_c_ kg^−1^, 300 cmol_c_ kg^−1^, and 750 cmol_c_ kg^−1^, respectively (Table 4).

**Figure 4:**
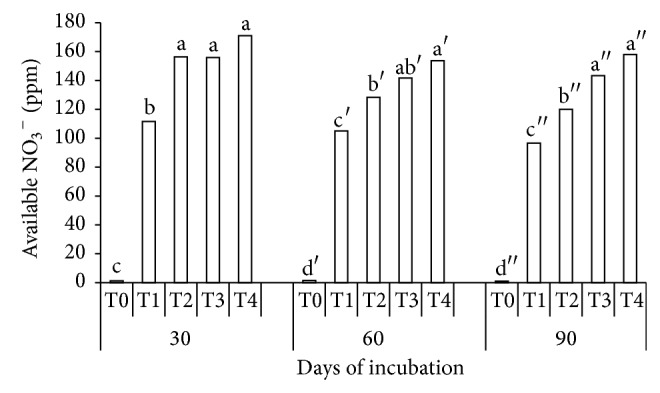
Effects of treatments (T0, T1, T2, T3, and T4) and periods (30, 60, and 90 days) of incubation on soil available nitrate.* Note*. T0: 250 g soil (no urea); T1: 250 g soil + 7.40 g urea (no additives); T2: 250 g soil + 7.40 g urea + 6 g clinoptilolite zeolite; T3: 250 g soil + 7.40 g urea + 6 g compost; T4: 250 g soil + 7.40 g urea + 6 g compost + 6 g clinoptilolite zeolite. Means with the same letter are not significantly different by Tukey's test at *P* ≤ 0.05.* Note*. Letters without prime represent 30 DAI, single prime superscript represents 60 DAI, and double prime superscript represents 90 DAI.

All the mixtures (T2, T3, and T4) significantly increased soil available NO_3_^−^ at 30 DAI, 60 DAI, and 90 DAI compared with urea without additives (T1) as demonstrated in Figure 4. The compost reduced leaching of NO_3_^−^ from the soil because of its C/N ratio (15.17) (Table 2). As reported by Kristensen et al. [48], incorporation of N rich compost, (low C/N ratio composts) led to rapid mineralization with associated increase in soil mineral N. At C/N ratio of 15 or less, mineralization occurs, whereas above a C/N ratio of 15, N is immobilized [48]. In a related study in which biosolids-yard waste compost was used to hinder NO_3_^−^ leaching, Xia et al. [49] reported that the concentrations of NO_3_^−^-N in their first leachates were high but they decreased in the subsequent leachates for all compost amended media. The higher soil available NO_3_^−^ at 30 DAI, 60 DAI, and 90 DAI in T2 and T4 compared with urea alone (T1) as presented in Figure 4 was because of the presence of clinoptilolite zeolite. This is possible because of the clinoptilolite zeolite's ion exchange system which enables absorption of anions such as NO_3_^−^ and phosphates [10]. The significant increase in the soil available NO_3_^−^ at 30 DAI, 60 DAI, and 90 DAI in T4 Figure 4 was also partly because of the increase in soil pH (due to pH of the compost). It is widely accepted that high pH has significant effect on availability of NO_3_^−^ as it influences nitrification and denitrification. Loss of NO_3_^−^ to N_2_O and NO emissions increase under low soil pH [45].

### 3.4. Leaching of Ammonium and Nitrate at Three-Day Interval

**Figure 5:**
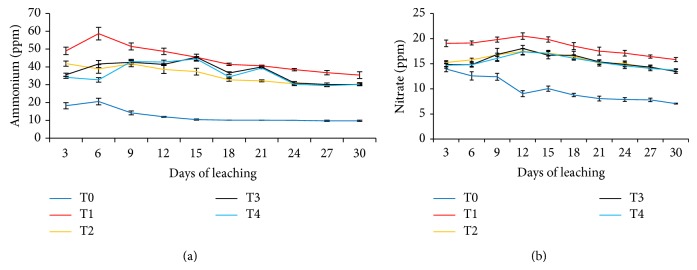
Ammonium and nitrate leached at three-day interval over 30 days of leaching experiment.* Note*. T0: 64 g soil (no urea); T1: 64 g soil + 7.40 g urea (no additives); T2: 64 g soil + 7.40 g urea + 6 g clinoptilolite zeolite; T3: 64 g soil + 7.40 g urea + 1.54 g compost; T4: 64 g soil + 7.40 g urea + 1.54 g compost + 6 g clinoptilolite zeolite.* Note*. Bar represents standard error of the mean.

The three-day interval losses of NH_4_^+^ and NO_3_^−^ from soil in the leaching experiment for 30 days are presented in Figures 5(a) and 5(b). As summarized in Figures 5(a) and 5(b), all of the mixtures (T2, T3, and T4) significantly reduced leaching of NH_4_^+^ and NO_3_^−^ from soil compared with soil alone (T0) and urea alone (T1). Treatments 2 and 4 (mixtures of urea and clinoptilolite zeolite) reduced NH_4_^+^ leaching because the clinoptilolite zeolite has high affinity for NH_4_^+^ (adsorption of NH_4_^+^ in the mineral lattices of clinoptilolite zeolite) whereas NO_3_^−^ was absorbed into the channels of the clinoptilolite zeolite. Huang and Petrovic [50] found that application of clinoptilolite zeolite to a sandy soil reduced NH_4_^+^ and NO_3_^−^ concentrations in leachate and increased moisture retention in the soil due to increased soil surface area and CEC. Thus, applying clinoptilolite zeolite to soils may reduce leaching of NH_4_^+^ and NO_3_^−^. The significant reduction in leaching of NH_4_^+^ and NO_3_^−^ in T2 and T4 reported in this study (Figures 5(a) and 5(b)) was comparable with those reported by Zwingmann et al. [51] whereby in a column experiment, clinoptilolite zeolite was used to reduce N leaching losses in a sandy soil.

**Figure 6:**
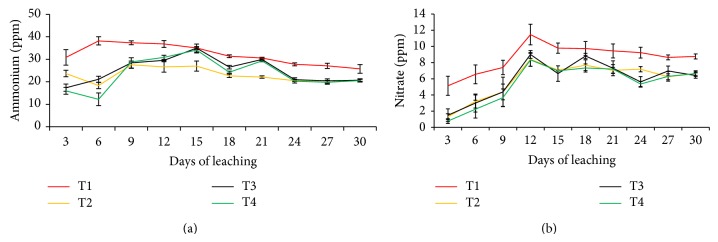
Ammonium and nitrate leached at three-day interval over 30 days of leaching for T1, T2, T3, and T4 effects only.* Note*. T1: 64 g soil + 7.40 g urea (no additives); T2: 64 g soil + 7.40 g urea + 6 g clinoptilolite zeolite; T3: 64 g soil + 7.40 g urea + 1.54 g compost; T4: 64 g soil + 7.40 g urea + 1.54 g compost + 6 g clinoptilolite zeolite.* Note*. Bar represents standard error of the mean.

For T3 and T4, leaching of NH_4_^+^ and NO_3_^−^ was reduced (Figures 5(a) and 5(b)) because of the affinity of the functional groups such as carboxyl and phenolic in the compost for NH_4_^+^ and NO_3_^−^. An evidence of this is the high contents of these functional groups of the humic acid (Table 4). The striking effects of urea amended with clinoptilolite zeolite and compost (T2, T3, and T4) on leaching of NH_4_^+^ and NO_3_^−^ compared with urea alone (T1) are clearly demonstrated in Figures 6(a) and 6(b) whereby the contribution of T0 (soil alone) was deducted from those of T1, T2, T3, and T4 over the 30 days of leaching experiment (i.e., leaching losses of NH_4_^+^ and NO_3_^−^ from T1, T2, T3, and T4 only).

### 3.5. Cumulative Losses of Ammonium and Nitrate for Thirty Days of Soil Leaching

**Figure 7:**
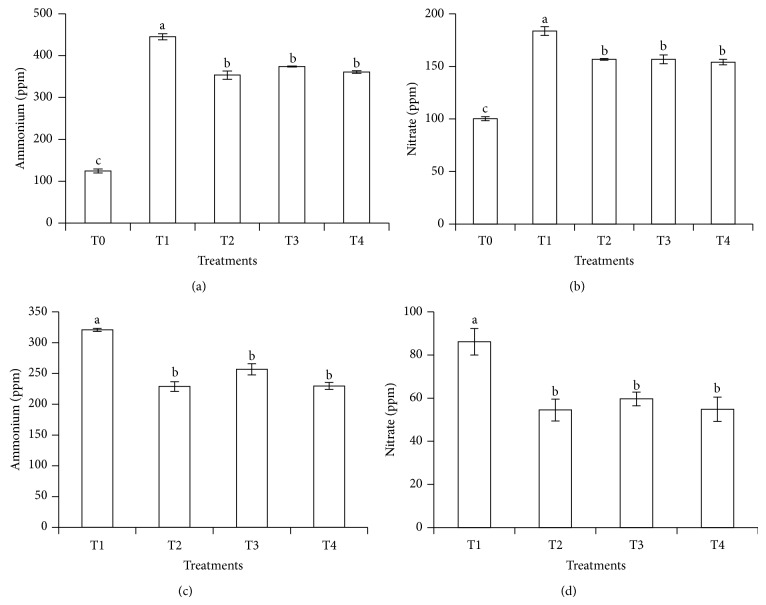
Cumulative amounts of ammonium and nitrate leached over 30 days of leaching experiment (a, b). Cumulative amounts of ammonium and nitrate leached (after subtracting the contribution of T0) over 30 days of leaching experiment (c, d).* Note*. T0: 64 g soil (no urea); T1: 64 g soil + 7.40 g urea (no additives); T2: 64 g soil + 7.40 g urea + 6 g clinoptilolite zeolite; T3: 64 g soil + 7.40 g urea + 1.54 g compost; T4: 64 g soil + 7.40 g urea + 1.54 g compost + 6 g clinoptilolite zeolite. Means with different letters indicate significant difference between treatments by Tukey's test at *P* ≤ 0.05.* Note*. Bar represents standard error of the mean.

Another evidence to support the effectiveness of T2, T3, and T4 in controlling NH_4_^+^ and NO_3_^−^ leaching loss compared with T1 is presented in Figure 7. All the treatments with clinoptilolite zeolite and compost (T2, T3, and T4) significantly decreased NH_4_^+^ and NO_3_^−^ leaching losses compared with urea without additives (T1). Treatments 3 and 4 significantly minimized NH_4_^+^ and NO_3_^−^ leaching losses partly because the compost served as a source of organic matter for N stabilization in the soil. This is because N is stored in soils in organic form; thus the quantity and nature of organic matter and its decomposition in the soil have effect on the long-term availability of N. The clinoptilolite zeolite in T2 and T4 was able to retain NH_4_^+^ because of the specific selectivity of the clinoptilolite zeolite for NH_4_^+^ [9]. This is one of the reasons why clinoptilolite zeolite is widely used as absorbent agent to capture N, after which the captured N is stored and released slowly for plant use [47].

### 3.6. Retention of Soil Exchangeable Ammonium and Available Nitrate at Thirty Days of Leaching Experiment

**Figure 8:**
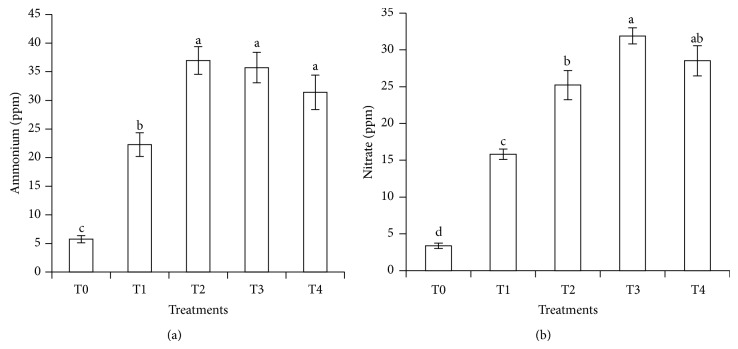
Retention of soil exchangeable ammonium and available nitrate after 30 days of leaching experiment.* Note*. T0: 64 g soil (no urea); T1: 64 g soil + 7.40 g urea (no additives); T2: 64 g soil + 7.40 g urea + 6 g clinoptilolite zeolite; T3: 64 g soil + 7.40 g urea + 1.54 g compost; T4: 64 g soil + 7.40 g urea + 1.54 g compost + 6 g clinoptilolite zeolite. Means with different letters indicate significant difference between treatments by Tukey's test at *P* ≤ 0.05.* Note*. Bar represents standard error of the mean.

At the end of the leaching experiment (30 days of leaching), urea with clinoptilolite zeolite and compost (T2, T3, and T4) showed significant concentrations of soil exchangeable NH_4_^+^ and available NO_3_^−^ compared to urea without additives (T1) (Figure 8). Soil exchangeable NH_4_^+^ and available NO_3_^−^ were lower in urea without additives (T1) at the end of leaching study because they were leached as discussed previously (Figure 7). The availability of NH_4_^+^ and NO_3_^−^ in the soil with clinoptilolite zeolite is possible because the channels in clinoptilolite zeolite effectively absorbed NH_4_^+^ and NO_3_^−^ and released them slowly. The high affinity of the clinoptilolite zeolite for NH_4_^+^ in particular is due to the small size of channels that protect NH_4_^+^ from excessive nitrification [9]. In a related study, clinoptilolite zeolite was used to improve soil retention of NH_4_^+^. The use of clinoptilolite zeolite also minimized the conversion of NH_4_^+^ to NO_3_^−^ [16].

The compost in T3 and T4 improved retention of soil exchangeable NH_4_^+^ and available NO_3_^−^ compared with urea without additives (T1) partly due to the ability of the compost to increase water holding capacity of soils. Due to this, leaching of NH_4_^+^ and NO_3_^−^ is reduced. This is possible because leaching losses of N occur when soils have more incoming water than the soil can hold. It was also reported that the slow release nature of compost-N renders leaching of NO_3_^−^-N [52]. As reported by numerous researchers [53, 54], application of compost to soils improved soil physical properties by increasing water holding capacity and porosity.

### 3.7. Soil Total Nitrogen after the Leaching Experiment

**Figure 9:**
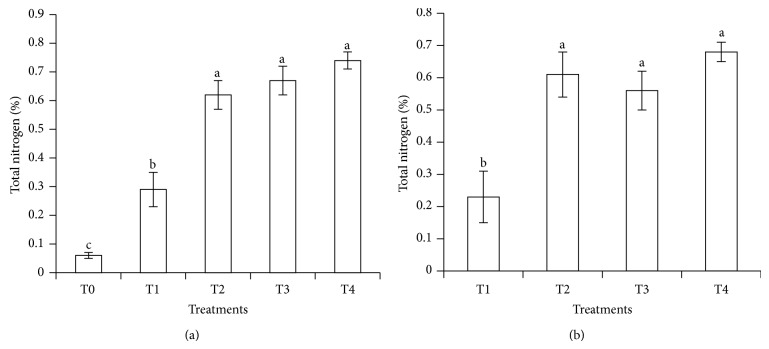
(a) Total nitrogen retained in soil at 30 days of leaching and (b) total nitrogen retained in soil after 30 days of leaching (after subtracting the contribution of T0).* Note*. T0: 64 g soil (no urea); T1: 64 g soil + 7.40 g urea (no additives); T2: 64 g soil + 7.40 g urea + 6 g clinoptilolite zeolite; T3: 64 g soil + 7.40 g urea + 1.54 g compost; T4: 64 g soil + 7.40 g urea + 1.54 g compost + 6 g clinoptilolite zeolite. Means with different letters indicate significant difference between treatments by Tukey's test at *P* ≤ 0.05.* Note*. Bar represents standard error of the mean.

The soil total N after the leaching experiment without subtracting the contribution of T0 from T1, T2, T3, and T4 is shown in Figure 9(a) and after subtracting the contribution of T0 is presented in Figure 9(b). The higher concentrations of soil total N in T2, T3, and T4 (urea with clinoptilolite zeolite and compost) than in T1 suggest that both clinoptilolite zeolite and compost ensured slow release of urea-N. For T1 (urea alone), retention of total N was lower compared with urea with clinoptilolite zeolite and compost (T2, T3, and T4) because N was lost due to leaching of NH_4_^+^ and NO_3_^−^ as discussed previously in Figures 5, 6, 7, and 8.

### 3.8. The pH of Leachate over 30 Days of Leaching

**Figure 10:**
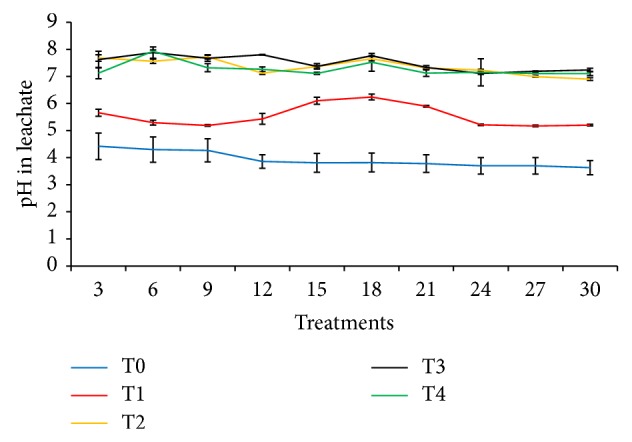
pH in leachate at three-day interval over 30 days of leaching experiment.* Note*. T0: 64 g soil (no urea); T1: 64 g soil + 7.40 g urea (no additives); T2: 64 g soil + 7.40 g urea + 6 g clinoptilolite zeolite; T3: 64 g soil + 7.40 g urea + 1.54 g compost; T4: 64 g soil + 7.40 g urea + 1.54 g compost + 6 g clinoptilolite zeolite.* Note*. Bar represents standard error of the mean.

The pH of leachate over 30 days of leaching is summarized in Figure 10. The lower pH in T1 (urea alone) compared with the treatments with clinoptilolite zeolite and compost (T2, T3, and T4) observed in this study explains the loss of N as discussed previously. The pH of leachate in T1 was lower from the third day of the leaching experiment and stayed above pH 6 for 15, 18, 21, and 24 days after which the pH was below 6 towards the end of leaching experiment. The formation of dissolved NH_3_ could be the reason for lower leachate pH in T1. This is because H^+^ released from NH_4_^+^ lowered pH of the leachate. The ability of T2 and T4 to maintain the pH of the leachates was because of the buffering capacity of clinoptilolite zeolite and compost. In a related study, Prasad and Foster [52] stated that acidification processes can be balanced by maintaining or enhancing pH through regular compost use. Only few experiments have led to a pH decrease after compost application [52].

## 4. Conclusion

Urea amended with clinoptilolite zeolite or compost, or combination of clinoptilolite zeolite and compost, reduced nitrogen (leaching of ammonium and nitrate) loss by retaining ammonium and nitrate in soil. The findings in this present study suggest that urea can be properly managed if it is amended with clinoptilolite zeolite or compost or combination of clinoptilolite zeolite and compost. Field application of our findings is being evaluated in our ongoing field experiment.

## Figures and Tables

**Table 1:** Selected chemical properties of Bekenu Series.

Property	Value obtained	Standard data range^*^
	(mean ± SE)	
CEC (cmol_c_ kg^−1^)	7.43 (±0.15)	8.0–24
pH_water_	4.66 (±0.10)	4.60
Exchangeable calcium (cmol_c_ kg^−1^)	1.41 (±0.05)	0.01
Exchangeable magnesium (cmol_c_ kg^−1^)	1.53 (±0.05)	0.21
Exchangeable potassium (cmol_c_ kg^−1^)	0.60 (±0.02)	0.19
Total nitrogen (%)	0.15 (±0.01)	0.04–0.17
Organic matter (%)	2.06 (±0.10)	nd
Total carbon (%)	1.20 (±0.60)	0.57–2.51
Available phosphorus (mg kg^−1^)	4.16 (±0.13)	nd
Exchangeable ammonium (mg kg^−1^)	19.85 (±0.68)	nd
Available nitrate (mg kg^−1^)	5.16 (±0.09)	nd

*Note*. ^*^Standard data range reported by Paramananthan [24]; nd = not determined.

Values in parenthesis represent standard error of the mean.

**Table 2:** Selected chemical properties of clinoptilolite zeolite.

Property	Present study	Reference^*^
	(mean ± SE)	
pH	6.80 (±0.03)	8-9
CEC (cmol_c_ kg^−1^)	100.33 (±0.35)	160
Total nitrogen (%)	1.18 (±0.04)	1.36
Calcium (mg kg^−1^)	18,400 (±19.09)	25,600
Magnesium (mg kg^−1^)	11,200 (±4.48)	15,000
Potassium (mg kg^−1^)	14,850 (±10.17)	22,600
Ammonium (mg kg^−1^)	12.60 (±0.43)	nd
Nitrate (mg kg^−1^)	11.58 (±0.18)	nd

*Note*. CEC = cation exchange capacity, nd = not determined. ^*^Data were obtained from Luxurious Empire Sdn. Bhd., Kulaijaya, Malaysia. Values in parenthesis represent standard error of the mean.

**Table 3:** Selected chemical properties of compost by cocomposting rice straw and chicken manure.

Property	Value obtained^*^ (mean ± SE)
pH value	7.66 (±0.07)
Humic acid (%)	15.20 (±0.32)
EC (ds m^−1^)	1.15 (±0.02)
Total carbon (%)	27.32 (±0.42)
Organic matter (%)	47.09 (±0.73)
Total nitrogen (%)	1.80 (±0.06)
C/N ratio	15.17^**^
Ammonium (mg kg^−1^)	294 (±2.84)
Nitrate (mg kg^−1^)	161 (±5.23)
Total phosphorus (mg kg^−1^)	458.20 (±5.50)
Calcium (mg kg^−1^)	14,080 (±6.91)
Magnesium (mg kg^−1^)	15,350 (±3.92)
Potassium (mg kg^−1^)	27,720 (±2.72)
Iron (mg kg^−1^)	13.10 (±0.42)
Zinc (mg kg^−1^)	11.80 (±0.15)
Copper (mg kg^−1^)	12.40 (±0.21)
Manganese (mg kg^−1^)	2.10 (±0.05)

*Note*. ^*^Values were obtained from our previous study on cocomposting rice straw and chicken manure (paper under review). Values in parenthesis represent standard error of the mean. ^**^Carbon to N ratio was calculated by dividing the percentage of C by the percentage of N.

**Table 4:** Selected chemical properties of humic acid extracted from compost.

Property	Value obtained (mean ± SE)	Tan^*^ [25]
E_4_/E_6_	7.73 (±0.07)	7-8
Phenolic (cmol_c_ kg^−1^)	300 (±6.42)	240–540
Carboxyl (cmol_c_ kg^−1^)	450 (±10.39)	150–440
Total acidity (cmol_c_ kg^−1^)	750 (±8.08)	500–700

*Note*. Values in parenthesis represent standard error of the mean. ^*^Value obtained from previous study [25].

**Table 5:** Significant levels from analysis of variance (ANOVA) to determine the effects of treatments and time on total N, soil exchangeable ammonium, and available nitrate.

Source of variation	Degree of freedom	Mean square		
		Total N	NH_4_^+^	NO_3_^−^
Time	2	0.291^*^	15728.8^*^	2625.4^*^
Replication	2	0.002^ns^	132.9^ns^	152.9^ns^
Error a (time ∗ replication)	4	0.001^ns^	126.7^ns^	57.4^ns^
Treatment	4	0.080^*^	79170.1^*^	33608.7^*^
Time ∗ treatment	8	0.004^*^	1543.4^*^	282.5^*^
Error b	24	0.001	112.7	73.1

*Note*. ∗ indicates significance at *P* ≤ 0.05. ns indicates no significance.
